# Effects of Six Commercial* Saccharomyces cerevisiae* Strains on Phenolic Attributes, Antioxidant Activity, and Aroma of Kiwifruit (*Actinidia deliciosa* cv.) Wine

**DOI:** 10.1155/2017/2934743

**Published:** 2017-01-30

**Authors:** Xingchen Li, Yage Xing, Lin Cao, Qinglian Xu, Shaohua Li, Ranran Wang, Zijing Jiang, Zhenming Che, Hongbin Lin

**Affiliations:** Sichuan Province Key Laboratory of Food Biotechnology, College of Food and Bioengineering, Xihua University, Chengdu 610039, China

## Abstract

“Hayward” kiwifruit (*Actinidia deliciosa* cv.), widely planted all around the world, were fermented with six different commercial* Saccharomyces cerevisiae* strains (BM4×4, RA17, RC212, WLP77, JH-2, and CR476) to reveal their influence on the phenolic profiles, antioxidant activity, and aromatic components. Significant differences in the levels of caffeic acid, protocatechuate, and soluble solid content were found among wines with the six fermented strains. Wines fermented with RC212 strain exhibited the highest total phenolic acids as well as DPPH radical scavenging ability and also had the strongest ability to produce volatile esters. Wines made with* S. cerevisiae* BM 4×4 had the highest content of volatile acids, while the highest alcohol content was presented in CR476 wines. Scoring spots of wines with these strains were separated in different quadrants on the components of phenolics and aromas by principal component analyses. Kiwifruit wines made with* S. cerevisiae* RC212 were characterized by a rich fruity flavor, while CR476 strain and WLP77 strain produced floral flavors and green aromas, respectively. Altogether, the results indicated that the use of* S. cerevisiae* RC212 was the most suitable for the fermentation of kiwifruit wine with desirable characteristics.

## 1. Introduction

“Hayward” kiwifruit (*Actinidia deliciosa* cv.) is one of the most popular fruits today and is cultivated extensively in New Zealand, China, the United States, and Southern Europe [1]. The popularity of kiwifruit should be not only due to its fresh flavor and succulent mouth-feel, but also because of its high content of vitamin C, vitamin E, phenolics, and other bioactive compounds that have high antioxidant effects and are beneficial for overall health [2, 3]. However, further development of the kiwifruit industry and economy has been significantly limited by the short shelf-life of the fruit and frequent losses during storage [4]. The extremely concentrated production period of the fruit contributes to poor-quality fruits, accelerated senescence, and short storage life [5, 6]. Making wine with postharvested kiwifruit can mitigate these problems by cutting down on waste. More importantly, kiwifruit wine is soft and mellow with unique flavor, and there is no doubt that its popularity will continue to grow around the world in the future, especially in places like China, where the fruit is already plentiful [7].

The bioactive compounds and flavor quality of kiwifruit wine are affected by many factors including brewing technology, fermentation culture, origin, raw materials, and aging [8, 9].* Saccharomyces cerevisiae*, as the key yeast in the microorganism for fermentation, is one of the most important contributor for the aroma, flavor, and bioactive components of fruit wine [10, 11]. Li et al. [12] found that different yeast strains have notable influence on polyphenol content during cider fermentation. Sun et al. [13] also revealed that cherry wines fermented with* S*.* cerevisiae* BM4×4 retained the highest content of phenolic acids, while* S*.* cerevisiae* D254 wines kept the lowest phenolic acids and higher terpene content. Among Riesling wines,* S. cerevisiae* EC1118, V1116, and VL1 yeast strains showed a definite impact on the odor-active compounds [14]. These results altogether suggested that the application of different strains of* S. cerevisiae* during wine fermentation could significantly influence the bioactive components (e.g., phenolics and polysaccharides), aroma constituents, and sensory characteristics of the wine [15, 16].

Phenolic compounds are some of the most important active substances that determine the character of kiwifruit wine. These compounds are also of interest to consumers due to high antioxidant levels and antimicrobial activity [17, 18], greatly contributing to the sensory properties of the wine by affecting its color and taste [19, 20]. Most of these phenolic compounds originate from fresh fruit to the wine during prefermentation, and a few are newly formed during the fermentation process [21]. Each individual yeast strain differed in adaptability, ethanol-production, and sugar-reduction ability [14]. Because of this, both the contents of phenolic substances and antioxidant activity in the wine system had differences depending on the strain that is used [13, 22]. Therefore, the selection of yeast strains is directly related to the composition and content of phenolics when the fermentation process is fully consistent.

Aroma is one of the key factors in distinguishing the character of fruit wines as it significantly affects the flavor and quality of the wine system [23]. The first-level aromas of fruit wines are terpene compounds and isoprene derivatives determined by the variety and origin of the fruit. Second-level aromas are produced during fermentation and are significantly affected by the yeast [10, 24]. The function of yeast is to release flavor and synthesize varietal volatile compounds [16]. So, finding a suitable yeast strain is crucial as it can produce the most desirable fermentative aromas, such as ethyl and acetate esters that provide fruity or floral nuances [11, 25]. To date, only the fermentation and fruit quality of kiwifruit wine have been widely investigated by researchers. No paper has yet been published on the effects of different commercial* S. cerevisiae* strains on the phenolic attributes, antioxidant activity, or aroma of kiwifruit wine.

The first objective of this study was to investigate the effects of different commercial* S. cerevisiae* on the phenolic profiles and antioxidant activity in kiwifruit wines. The further purpose of this study was to identify and quantify volatile aromas of kiwifruit wines fermented with* S. cerevisiae* strains in terms of producing wine with a unique flavor profile and pleasant character.

## 2. Materials and Methods

### 2.1. Chemicals

Phenolic standards, volatile standards, Folin-Ciocalteu's phenol reagent, 2,2-diphenyl-1-picrylhydrazyl free radical (DPPH), 6-hydroxy-2,5,7,8-tetramethyl chroman-2-carboxylic acid (Trolox), 2,2′-azino-bis and 3-ethylbenzothiazoline-6-sulfonic acid (ABTS), ethanol (chromatographic grade), and methanol (99%) were purchased from Sigma-Aldrich (St. Louis, MO, USA). Other routine chemicals were purchased from Aobo Chemical Co. (Beijing, China).

### 2.2. Kiwifruit Sample Preparation

“Hayward” kiwifruits (*Actinidia deliciosa cv.*) with the soluble solid content of 16°Brix were harvested from orchards in Cangxi county (32°10′N, 105°45′E), Sichuan province, China, which were used as the material for the wine production in this research. The harvested fruits were chosen with absence of disease infection or physical injuries firstly and then immediately stored at 4°C in the cool room of Fruits & Vegetables Preservation Laboratory in Xihua University for further studies.

### 2.3. Fermentation Technology of Kiwifruit Wine

#### 2.3.1. Yeast Strains

Six commercial strains of* S. cerevisiae* were selected and purchased from Lallemand (France) in this study, which are Lalvin BM4×4, RA17, RC212, WLP77, JH-2, and CR476, respectively [12, 13]. These strains showed adaptive characteristics, such as good fermentation speed, low production of foam, and growth at high or low temperature [26], maintained separately at 4°C on yeast extract peptone dextrose agar medium (2% glucose, 2% peptone, 1% yeast extract, and 2% agar), as well as in glycerol stocks at −80°C. Yeast strains should be activated and cultured at 28°C before the fermentation procedure. The commercial yeast, Zyma F15 (LAFFORT, France), applied with good results in some Chinese wineries, was used as the Control.

#### 2.3.2. Prefermentation Procedures

The prefermentation procedures of kiwifruit wine were conducted according the method reported by Wang et al. [7] with some modifications. The kiwifruits were sorted by size and quality, then manually peeled, and pulped. Pectinase (0.15 g/l, activated: 10000 U/g) and SO_2_ (40 mg/L) were then added to the juice. Kiwi juice was obtained by centrifugation of fruit pulp (speed: 4000 rpm/min, time: 6 min) and placed into a fermentation jar [27]. Finely granulated sugar was then mixed into hot water at a ratio of 1 : 2 and stirred; then the syrup was poured into the fermentation jar until the sugar content of the kiwi juice was 20°Brix. The mixture was then set aside to cool.

#### 2.3.3. Fermentation

Precultures for the six* S. cerevisiae* yeasts were used to inoculate kiwifruit juice blends at a final concentration of 10^6^ CFU/mL. The six samples were fermented at 25 ± 1°C for two weeks. The fermented samples were then racked into a secondary jar and further aged under the same conditions. The whole fermentation process was monitored by measuring the soluble solid content (SSC). After four weeks, the fermentation was stopped by adding the SO_2_ (50 mg/L) to the samples until the SSC contained less than 8°Brix. The finished young kiwi wine was filtered through filter plates, then bottled, and stored at 16°C until subsequent analysis [15]. All fermented samples were completed in quadruplicate.

### 2.4. Conventional Analysis

The soluble solid content (SSC) was determined by a refractometer (Model 0–35°Brix, Jiahuang Instruments, China). Titratable acidity (TA) was measured by titrating a sample (4 mL of juice or wine diluted with 20 mL of distilled water) with 0.1 N NaOH. Alcohol content (% v/v) was analyzed using the Gay Lussac Table by distilling and adjusting 100 mL of fermented sample to 15°C. The pH was measured with a Thermo Orion 420 at pH meter (Thermo Fisher Scientific Waltham, MA, USA). The transmittance (*T*%) was measured by ultraviolet-visible 7200 spectrophotometer (Unico Instrument Co., Shanghai, China) with colorimetric cup, and distilled water was used as blank. The values for CIE* L*, CIE *a*, and CIE *b* were determined with CR-400 Chroma meter equipped with CR-S4w utility software (Konica Minolta Sensing Inc., Japan).

### 2.5. Phenolic Profiles

Phenolic compounds in the wine samples were determined with an Agilent 1230 HPLC system using methods described by Wang et al. [7] and Porgali and Büyüktuncel [28] with some modifications. The wine samples and standard solutions were filtered through a 0.2 *μ*m syringe filter and 20 *μ*L of the filtrate was injected into the HPLC system. Chromatographic separation was performed on a C18 reversed-phase Symmetry Analytical column (5 *μ*m × 250 mm × 4.6 mm; Waters Corp., Milford, MA). Two different mobile phases were prepared for this purpose: mobile phase A was 10 mM phosphoric acid solution and mobile phase B was methanol. The optimized gradient program for phase B was as follows: 0–15 min (0–60%), 15–20 min (60–80%), 20.0–22 min (80–100%), 22–27 min (100–0%), and 27–32 min (0%). Flow rate was 1 mL/min during analysis time and injection volume was 10 *μ*L. Detection wavelengths were determined according to the spectra obtained from Agilent Chem Station Software.

### 2.6. Total Phenolics and Antioxidant Activity

Total phenolic content of the wine samples was measured according to the method described by Ivanova et al. [29] with some modifications. The results were expressed as milligrams of gallic acid equivalents (GAE). Scavenging free radical potentials were tested in a solution of 1-diphenyl-2-picrylhydrazyl (DPPH). This DPPH solution (3.9 mL, 25 mg/L) in methanol was mixed with the sample extracts (0.1 mL), then the reaction process was monitored at 515 nm until the absorbance was stable [30]. The screening of antioxidant capacity was reported as a decolorization assay with 2,2-Azino-bis(3-ethyl-benzothiazoline-6-sulfonic acid) diammonium salt (ABTS) method [3, 31].

### 2.7. Aroma Analysis

#### 2.7.1. Aroma Analysis Method

For each SPME analysis, 10 mL of wine (7.0 g/L tartaric acid, 12% v/v), 1.8 g of NaCl, and an internal standard mixture (octyl propionate with a final concentration of 60.44 *μ*g/L, 3-octanol with a final concentration of 225.4 *μ*g/L) were placed in 20 mL vials, capped with a PTFE-silicon septum, and heated to 40°C. After 20 min of stirring at 1100 rpm/min, the SPME fiber (80 *μ*m PDMS; Supelco, USA) was exposed to the sample headspace for 40 min and then inserted into the GC injection port for a 3 min desorption time.

The analysis and GC-MS technology were operated according to a previous description by Rebière et al. [32]. The samples were analyzed on a 6890 N gas chromatograph (Agilent Technologies, Santa Clara, CA) using an Agilent HP-5 MSI column (5% phenylmethyl polysiloxane, 30 m, 0.25 mm i.d., 0.25 lm film thickness) in combination with a 5973 mass selective detector. Chromatographic conditions were inlet at 250°C with injection volume of 2 *μ*L in pulsed splitless mode. During the analysis period, the split ratio was 1 : 3 and flow rate (He) was 1 mL/min. The temperature program was from 40°C (0 min) to 140°C for 2 min followed by an increase to 250°C at a rate of 10°C/min and a 5 min hold, then from 250°C to 300°C at 15°C/min, and finally 300°C for 5 min. Other conditions included an ion source of 250°C, electron impact (EI) mode with an ionization voltage of 70 eV, and mass range of 35–350 amu/s.

#### 2.7.2. Qualitative and Quantitative Analyses

Compound identity was verified according to mass spectral library matches (NIST 05 Database, Agilent Technologies) when the matching degree was greater than 90%. The spectra, retention time, and aromatic characteristics of the existing standard compounds were confirmed [33]. Using the standard addition method, 26 volatile components were quantitatively analyzed and the contents of unknown compounds in the samples were obtained by extrapolation. The standard solutions were prepared by diluting the stock solution in synthetic wine (7.0 g/L tartaric acid in 12% alcohol solution) to obtain a range of concentrations. The calibration curve for each target compound was built by plotting the selected mass ion abundance ratio [34]. Quantitative data of the identified compounds were obtained by interpolation of the selected mass ion areas versus the internal standard area.

### 2.8. Sensory Analysis

Sensory analyses were conducted separately to determine both aromatic and tactile attributes. The aromatic attributes were determined according to odor descriptors and the tactile attributes developed by Gawel et al. [35]. These attributes of the wines were assessed by a sensory panel consisting of eight wine industry professionals (four women and four men) selected from staff and faculty members of Xihua University, China, who are familiar with wine sensory characteristics and with a minimum of three years of wine-tasting experience. We provided a small amount of training to explain the scoring methodology and defined the necessary terms. Wines were randomly served and water was provided for palate cleansing. The sensory attributes were scored on a ten-point scale with zero representing “nonexistent” and ten representing “extreme” [17].

### 2.9. Statistical Analysis

Samples were analyzed in quadruplicate for each wine replicate and results expressed as the mean value ± standard deviation (SD). The quadruplicate wine samples were statistically analyzed in SPSS 19.0 for Windows (SPSS Inc., Chicago, Illinois, USA) via analysis of variance (ANOVA) and Dunnett's multiple range tests. The figures of Principal Component Analysis (PCA) performed the phenolic profiles and aroma compounds also in Origin 8.6 (OriginLab, Hampton, Massachusetts) in order to determine the differences in data sets and to establish the relationships between samples and phenolic profiles as well as samples and volatile compounds.

## 3. Results and Discussion

### 3.1. Conventional Analysis of Kiwifruit Wine

The six* S. cerevisiae* yeasts (BM4×4, RA17, RC212, WLP77, JH-2, CR476, and the Control strain) were inoculated in the fruit juices under the fermentation conditions of kiwifruit wine at 25°C. The basic indexes were shown in Table 1, including pH, SSC, alcohol content (AC), titratable acidity (TA), and transmittance (*T*). The differences of pH values could be ignored in these wines. AC reflected the ability of yeasts to produce alcohol during the fermentation process [38]. A moderate amount of alcohol had significant differences (*P* < 0.01) among wines fermented with different yeasts, ranging from 8.5% v/v to 11.9% v/v. The differences in TA were significant across all wine samples. The lowest TA content was found in kiwifruit wine fermented with RA17 at 10.20 g/L and the highest with CR476 at 12.25 mg/L. The SSC of the wine was all 20°Brix before fermentation, wines with* S. cerevisiae* RC212 generated the lowest concentrations of 4.4°Brix at the end of fermentation. CIE *L* and CIE *b* differed significantly (*P* < 0.01). Wine fermented with RC212 showed the highest values of CIE* L*, which determines the intensity of the green color of the wine.

It was noted that all pH values in the kiwifruit wines were lower than that of cherry wine (4.07–4.22), red wine (3.59–3.66), or Sauvignon Blanc (2.97–3.09) [22, 26, 30]. This may have been caused by the different pH values in the raw fruits. The average pH of fresh kiwifruit is lower than that of most other fruits [39], which was preserved in the kiwifruit wine. Our results also indicate that the different strains of* S. cerevisiae* used in fermentation significantly affected the AC in the system. A drastic decrease in SSC further confirmed that the selected yeasts dominated the fermentation process [16]. SSC was lowest in RC212 regardless of fermentation time, suggesting that this strain had optimal adaptation and ability to reduce sugar. Wines made with RC212 retained the most ideal green color as reflected in the high CIE* L*, which was consistent with the color findings in cherry wines with this strain [26]. The color, transmittance, and clarity of kiwifruit wine are important parameters for consumer acceptance and are thus necessary for the production of high-quality fruit wines.

### 3.2. Phenolic Profiles in Kiwifruit Wines

**Figure 1:**
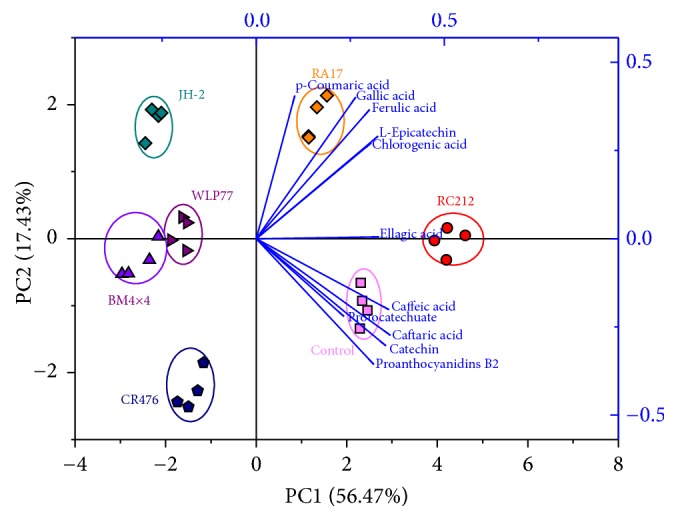
Principle component analysis biplot of the phenolic substances from kiwifruit wines with six* S. cerevisiae* commercial strains including BM4×4, RA17, RC212, WLP77, JH-2, and CR476.

Polyphenolic compounds are known to influence the color and flavor of wines and also play a major role in their nutrition and health benefits. As shown in Table 2, the highest content of total phenolic compounds in the wines fermented with* S. cerevisiae* RC212, followed by RA17 and BM4×4. Caffeic acid content (1.185–2.797 mg/L) differed significantly (*P* < 0.001) in all tested samples, reaching the highest levels in RC212 wines and the lowest in WLP77. The concentrations of L-epicatechin (0.748–1.961 mg/L) in wines fermented with RA17 and RC212 were significantly higher than that in the wines produced with other strains. Catechin content also differed significantly (*P* < 0.001) across all the samples (and was undetectable in BM4×4 and JH-2 wines). Proanthocyanidins B_2_ and gallic acid were also found in low levels. Additionally, the PCA analysis was performed on the phenolic attributes in effort to further understand how these differences impacted the quality of the wine (Figure 1).

As shown in Figure 1, the points of wines with different strains are scattered in different areas. All of the phenolic compounds had a positive effect on the first PC (56.47%), while some compounds, including ellagic acid, caffeic acid, caftaric acid, catechin, protocatechuate, and proanthocyanidins B2, had negative effects on the second PC (17.43%). The RC212, BM4×4, and WLP77 wines were approximately located in the* x*-axis of PC2. The RC212 wines are located in the right hand quadrant and the BM4×4 and WLP77 wines are situated in the left. Wines from JH-2 are situated in the upper area of BM4×4 wines, while CR476 wines are located below. The same figure shows phenolic compounds correlating with individual wine treatments: wines from the RA17 group were correlated with* p*-coumaric acid, ferulic acid, gallic acid, and chlorogenic acid, while Control wines were correlated with caffeic acid, caftaric acid, catechin, and proanthocyanidins B_2_. The wines from RC212 were particularly associated with ellagic acid.

The above results indicated different* S. cerevisiae* strains had close relationship with differences in individual polyphenolic compounds, which is consistent with previous studies conducted by Loira et al. and Yanlai et al. [14, 27]. Most of these phenolic compounds passed from fruit to wine remained active, but their profiles exhibit varying degrees of change and degradation. This causes differences in physical and chemical reactions and leads to different structures and concentrations of phenolic compounds in the wine [40]. Undoubtedly, the choice of yeast strain dramatically influences phenolic composition and contents. Beyond this, the chemical structures and properties of phenolic compounds are susceptible to other factors including pH changes, enzymatic reactions, vegetation season, maturity of fruits, and fermentation conditions [20, 30, 41]. In sum, the difference of phenolic compounds in wines fermented with BM4×4 and WLP77 was negligible and classed as one group. Kiwifruit wines with other strains were clustered into a separate quadrant of the biplot. More importantly, RC212 strain and RA17 strain are capable of the retention and production of individual phenolics.

### 3.3. Total Phenolics and Antioxidant Activity in Kiwifruit Wines

The total phenolics and antioxidant activity in kiwifruit wines also were determined and evaluated. As shown in Table 2, we observed significant differences among all sample wines (*P* < 0.01). Total phenolics content varied from 234 mg GAE/L in RC212 fermented wines to 317 mg GAE/L in CR476 wines. This suggests that RC212 resulted in the highest extraction of total phenolics from kiwifruit flesh and skins, and there was a significant increase in antioxidants in wines made with RC212 compared to that in the Control wines. The results of ABTS scavenging capacity was nearly consistent with the analysis of DPPH scavenging capacity. In short, the differences of wines with* S. cerevisiae* strains were revealed on total phenolics and antioxidant activity. Loira et al. similarly observed a variation in total phenolic content in red wines with different strains [14]. Czyzowska and Pogorzelski [42] found high antioxidant activity in wines containing high amounts of total phenolics, and other researchers have revealed positive correlations between total phenolic content and antioxidant capacity [30, 43]. These results were in line with our finding that RC212 wines not only contained the highest total phenolics, but also showed the greater DPPH radical scavenging activity, which means that* S. cerevisiae* RC212 had an excellent ability to preserve total phenolics and antioxidants in kiwifruit wines.

### 3.4. Aroma in Kiwifruit Wines

#### 3.4.1. Analysis of Aroma Compounds in Kiwifruit Wines

As shown in Table 3, the concentrations of wine aroma compounds and odor descriptors differed substantially among the samples. A total of 26 compounds were identified and quantified in all wines, including seven higher alcohols, four acetates, nine acetate esters, four acids, and one ketone. The highest content of total volatile compounds was found in RC212, followed by WLP77, BM 4×4, and RA17. Among the 13 esters quantified, ethyl caprylate has the highest average content of esters, standing out (*P* < 0.01) among wines with diverse strains. Concentrations of ethyl cinnamate, a cinnamon flavor, differed significantly (*P* < 0.001) compared to the Control samples. As shown in Table 3, the majority of the esters contained unique flavor characteristics, such as methyl butyrate (apple, banana, and pineapple), ethyl butyrate (pineapple, floral), and ethyl laurate (sweet, fruity). Yeast RC212 had the largest concentration of total volatile esters (13.65 mg/L), nearly three times higher than those contained in JH-2. Alcohols were quantitatively the second largest group of the flavor compounds in our wines. Isopentyl alcohol had a laurel oil flavor and was found in the greatest quantity in CR476. The amount of pentyl alcohol (*P* < 0.001) differed among all strains. These results indicate that CR476 produced more alcohol, generating the highest content of total volatile alcohols (11.48 mg/L). Kiwifruit wines also contain more acids than most fruit wines [7]. The concentration of volatile acids in kiwifruit wines was about 1.2 times that in cherry wines and 1.5 times as much as in ciders [7, 11]. However, acids usually affect pleasant aroma negatively [27]. Octanoic acid produced an undesirable cheesy odor, and both acetic acid and isobutyric acid had a sour and rancid flavor (Table 3). RC212 was characterized in general by a substantial amount of aroma compounds and esters, while CR476 and BM 4×4 had the highest amount of alcohol and volatile acids, respectively.

Aroma compounds form the typical and special odor characteristics of wines, and* S. cerevisiae* is one of the most important factors affecting volatile composition for wine [11]. Our results demonstrated that different strains of* S. cerevisiae* lead to different compounds and concentrations of esters, alcohols, and acids in the wine system. Other strains of* S. cerevisiae* have also been reported to influence the aromatic profiles of wines [12, 20, 24]. These differences of aroma compounds may have been due to the fact that the main origin of these aroma compounds is yeast metabolism during fermentation [44] or possibly due to the interaction between biochemical mechanisms of yeast strains and the complex compounds in the fruit juice [14]. Furthermore, it is well known that esters are desirable compounds for giving wines fruity or floral flavor, and the RC212 wines contained the largest amount of esters. Therefore, RC212 strain should be utilized for the fermentation of kiwifruit wine in order to enhance its fruity and floral features.

#### 3.4.2. PCA Analysis of Aroma Compounds in Kiwifruit Wines

**Figure 2:**
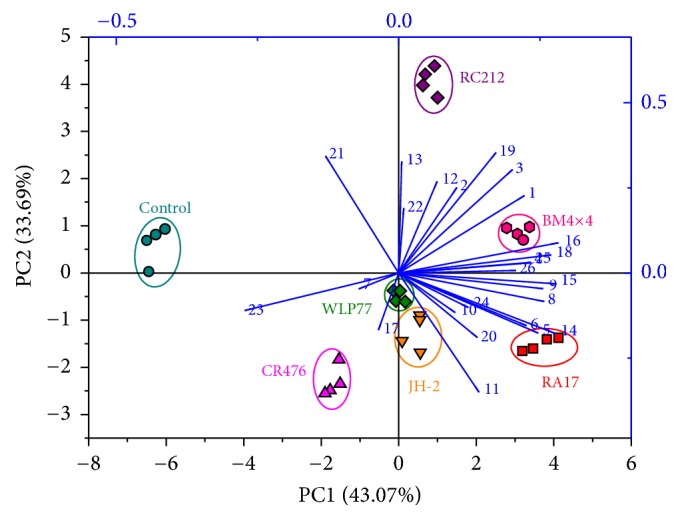
Principle component analysis biplot of aroma substances from kiwifruit wines with six* S. cerevisiae* commercial strains including BM4×4, RA17, RC212, WLP77, JH-2, and CR476.

The differences of aromatic profiles with PCA were further elucidated in wines fermented by different strains. The results in Figure 2 indicated that the first two PCs account for 76.76% of the variation in the aroma profiles. According to the biplot, most aroma components were positively correlated with the first PC (43.07%) with the exception of eugenol, isobutyric acid, hexyl acetate, and pentyl alcohol. Twelve aroma components were positively correlated with the second PC (33.69%) including eugenol, ethyl cinnamate, acetic acid, ethyl myristate, and methyl butyrate.

The spots of these seven types of kiwifruit wine were scattered in different quadrants. The RC212 wines and BM4×4 wines were spread in the first quadrant, which contributes to both the first and second PCs. The scores of RC212 wines were higher than those of BM4×4 in the first PC, but the scores of BM4×4 wines were higher in the second PC. The RA17 wines located in the lower right hand quadrant, highly correlating with the components of isobutyl alcohol and isoamyl acetate. The WLP77 wines and the JH-2 wines can both be found in the lower first PC, and CR476 wines distributed into the lower left quadrant. There was a close relationship between the specific aroma components and the different treatment groups in the same quadrant. In short, the PCA of different kiwifruit wines showed that single strains had considerable influence on the single aroma compounds that determine various characteristics of wines.

### 3.5. Sensory Analysis

**Figure 3:**
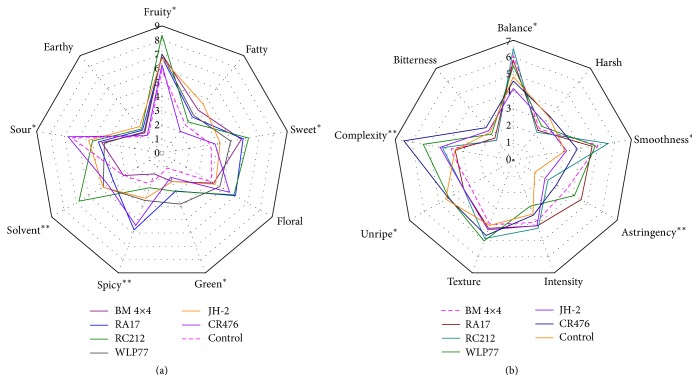
Average values of sensory evaluation scores from the panel. (a) Aroma. (b) Tactile attributes of quadruplicate wines per treatment. Significance determined by Dunnett's test with Control; ^*∗*^(*P* < 0.05) and ^*∗∗*^(*P* < 0.01).

As mentioned above, sensory analysis was broken into two separate groupings (aromas and tactile attributes). Several descriptors were statistically influenced by* S. cerevisiae* strains. As shown in Figure 3(a), significant differences (*P* < 0.01) in solvent and spicy flavors were found in the sample wines. Wines fermented with RA17 and CR476 exhibited more spicy attribute than other wines. The flavor of BM4×4 wines was similar to that of the Control samples. The WLP77 wines contained the highest evaluation about green flavor, while RC212 wines got the best assessment of fruity aroma. Wines fermented with CR476 were more sour than the others, possibly due to higher amounts of volatile acids (Table 1). According to the same analysis on tactile attributes, complexity and astringency (*P* < 0.01) were significantly different among the various sample wines. Wines produced by CR476 had the highest values of tactile complexity, followed by WLP77 and JH-2 wines. The application of RC212 significantly increased smoothness and balance in the fruit wine system; however JH-2 wines had the lowest score for these two attributes.

Aroma compound analysis results suggested that the application of different yeasts can indeed impact the odor profiles of kiwifruit wines. The kiwifruit wines scored particularly high on the fruity attribute, which coincided with high content of aroma compounds (e.g., isobutyl acetate, ethyl caproate, ethyl caprylate, and ethyl benzoate). Certain relationships observed between the sensory attributes and esters suggested that the fermentation period conditions induced chemical changes in the wine [45].

*S. cerevisiae* RC212 produced the highest concentrations of ethyl caproate, ethyl caprylate, ethyl decanoate, and ethyl benzoate, exhibiting the greatest amount of fruity odor.* S. cerevisiae* CR476 may be appropriate for wine preparation if the winemaker desires to bring out strong floral flavors, while* S. cerevisiae* WLP77 may be used to increase green aromas. In the next step, the combined use of RC212 strain and WLP77 strain in fermentation is ideal to provide unique fruit flavors and aromatic diversity for kiwifruit wine.

## 4. Conclusion

In this study, six* S. cerevisiae* strains for kiwifruit wine were evaluated in phenolic profiles, antioxidant activity, and volatile compounds for the sake of determining the ideal strain with favorable sensory qualities. The results indicated significant differences in caffeic acid and protocatechuate in the phenolic profiles of different strains. In regard to volatile compounds, wines with* S. cerevisiae* RC212 exhibited the highest total phenolic acid content and DPPH radical scavenging ability, and it also produced the highest quantity of volatile esters. Wines fermented with* S. cerevisiae* CR476 obtained the highest alcohol concentration and BM 4×4 had the highest volatile acid content. PCA results showed that the spots of phenolics and aroma compounds were scattered in different areas across the various kiwifruit wines. Sensory analysis also showed that kiwifruit wines with RC212 were characterized by an intense fruity flavor, while BM 4×4 wines showed acidic flavor. The yeast of* S. cerevisiae* CR476 enhanced the floral flavor of the wines, while WLP77 enhanced their green aromas. These results altogether demonstrated that the selection of* S. cerevisiae* strain affects the formation and concentrations of phenolics and volatile substances as well as the sensory quality of wines. The* S. cerevisiae* RC212 strain is likely the optimal choice for kiwifruit wine fermentation. The combined application of* S*.* cerevisiae* strains for kiwifruit wine merits further research.

## Figures and Tables

**Table 1:** Conventional analysis of kiwifruit wine (*n* = 4).

Wine	pH	SSC^*∗∗∗*^	AC^*∗∗*^	CIE *L*^*∗*^	CIE *a*^*∗*^	CIE *b*	TA^*∗*^	*T* ^*∗*^
Control	2.95 ± 0.03^a^	9.8 ± 0.5^a^	8.6 ± 0.7^a^	78.12 ± 1.03^a^	0.30 ± 0.02^a^	7.71 ± 0.41^a^	12.27 ± 0.10^a^	84.9 ± 1.2^a^
BM 4×4	3.03 ± 0.04^b^	7.4 ± 0.2^c^	10.2 ± 0.6^b^	80.96 ± 0.96^a^	0.27 ± 0.02^b^	7.78 ± 0.42^a^	12.14 ± 0.12^a^	86.4 ± 1.4^a^
RA17	2.97 ± 0.04^a^	5.5 ± 0.4^d^	11.0 ± 0.7^c^	82.37 ± 0.81^b^	0.24 ± 0.01^c^	7.54 ± 0.38^b^	10.20 ± 0.09^c^	88.3 ± 1.3^b^
RC212	2.95 ± 0.02^a^	4.4 ± 0.4^d^	11.9 ± 0.5^c^	88.12 ± 1.21^d^	0.32 ± 0.02^a^	7.41 ± 0.41^b^	10.37 ± 0.10^c^	91.3 ± 1.4^c^
WLP77	2.93 ± 0.03^a^	4.9 ± 0.3^d^	11.1 ± 0.7^c^	85.01 ± 0.98^b^	0.30 ± 0.00^a^	7.40 ± 0.30^b^	10.28 ± 0.11^c^	89.2 ± 1.2^c^
JH-2	2.91 ± 0.05^a^	8.3 ± 0.5^b^	8.5 ± 0.6^a^	77.10 ± 0.89^a^	0.23 ± 0.01^b^	7.55 ± 0.41^b^	11.00 ± 0.10^b^	85.5 ± 1.3^a^
CR476	2.97 ± 0.02^a^	6.7 ± 0.3^c^	10.5 ± 0.5^b^	79.31 ± 1.11^a^	0.25 ± 0.00^c^	7.40 ± 0.34^b^	12.25 ± 0.13^a^	87.2 ± 1.4^b^

Results were expressed as the mean of quadruplicates ± standard deviation (SD). Values with different superscript roman letters (a–f) in the same row are significant; *∗* represents significance at (*P* < 0.05), *∗∗* at (*P *< 0.01), and *∗∗∗* at (*P* < 0.001). Soluble solid content (SSC) values are performed by °Brix. Titratable acidity (TA) values and the alcohol content (AC) values are reported in g/L and % v/v, respectively. The transmittance (*T*) values are expressed with percentage.

**Table 2:** Phenolic concentrations in six kiwifruit wines, with corresponding total phenolics, ABTS radical scavenging activity, and DPPH radical scavenging activity (*n* = 4).

	CR476	JH-2	WLP77	RC212	RA17	BM 4×4	Control
Gallic acid^*∗∗*^	0.174 ± 0.021^c^	0.340 ± 0.034^a^	0.217 ± 0.023^c^	0.395 ± 0.018^b^	0.377 ± 0.035^ab^	0.291 ± 0.031^b^	0.351 ± 0.025^a^
Proanthocyanidins B2^*∗*^	0.359 ± 0.037^a^	0.207 ± 0.021^d^	0.322 ± 0.043^b^	0.451 ± 0.052^b^	0.287 ± 0.031^bc^	0.235 ± 0.027^d^	0.387 ± 0.032^a^
L-Epicatechin^*∗∗*^	0.748 ± 0.127^c^	1.243 ± 0.114^b^	1.554 ± 0.088^ab^	1.961 ± 0.134^ab^	1.829 ± 0.164^a^	0.802 ± 0.107^c^	1.763 ± 0.112^a^
Caffeic acid^*∗∗∗*^	1.786 ± 0.121^c^	1.055 ± 0.094^e^	1.185 ± 0.114^e^	2.797 ± 0.157^a^	2.011 ± 0.096^b^	1.564 ± 0.142^d^	2.636 ± 0.110^a^
Caftaric acid^*∗*^	0.576 ± 0.049^ab^	0.273 ± 0.042^d^	0.378 ± 0.074^c^	0.758 ± 0.051^b^	0.547 ± 0.037^b^	0.422 ± 0.023^c^	0.656 ± 0.047^a^
Ferulic acid^*∗*^	0.520 ± 0.054^a^	0.698 ± 0.085^b^	0.746 ± 0.073^b^	0.933 ± 0.088^c^	1.088 ± 0.124^c^	0.648 ± 0.057^ab^	0.971 ± 0.090^a^
Ellagic acid^*∗∗*^	0.265 ± 0.036^a^	0.214 ± 0.018^a^	ND	0.577 ± 0.019^c^	0.415 ± 0.031^b^	0.268 ± 0.021^a^	0.403 ± 0.022^a^
Catechin^*∗∗∗*^	0.276 ± 0.020^b^	ND	0.159 ± 0.010^d^	0.416 ± 0.026^b^	0.201 ± 0.019^c^	ND	0.347 ± 0.021^a^
Protocatechuate^*∗∗∗*^	0.135 ± 0.008^e^	0.206 ± 0.013^d^	0.291 ± 0.009^c^	0.645 ± 0.061^b^	ND	0.326 ± 0.032^c^	0.549 ± 0.024^a^
p-Coumaric acid	0.571 ± 0.030^a^	0.627 ± 0.022^b^	0.603 ± 0.031^a^	0.642 ± 0.050^b^	0.597 ± 0.039^a^	0.587 ± 0.041^a^	0.533 ± 0.059^a^
Chlorogenic acid	0.554 ± 0.042^b^	0.576 ± 0.033^b^	0.612 ± 0.044^a^	0.682 ± 0.035^a^	0.629 ± 0.048^ab^	0.453 ± 0.026^c^	0.649 ± 0.039^a^
Total phenolics^*∗∗*^	234 ± 11^d^	249 ± 7^c^	253 ± 13^c^	317 ± 10^b^	305 ± 15^ab^	272 ± 12^bc^	298 ± 11^a^
ABTS^*∗*^	13.49 ± 0.53^b^	11.63 ± 0.61^bc^	14.75 ± 0.58^ab^	16.05 ± 0.76^a^	15.98 ± 0.68^a^	12.33 ± 0.65^c^	15.89 ± 0.71^a^
DPPH^*∗∗*^	149 ± 12^bc^	126 ± 7^c^	131 ± 9^c^	201 ± 8^b^	193 ± 10^ab^	143 ± 11^bc^	178 ± 9^a^

ND: not detected.

Values with different superscript roman letters (a–d) in the same row are significantly different; *∗* represents significance at *(P* < 0.05), *∗∗* at (*P* < 0.01), and *∗∗∗* at (*P* < 0.0001). Phenolic content expressed in mg/L of standard. Antioxidant activity of DPPH radical scavenging and ABTS radical scavenging expressed in mg/L Trolox equivalents and Folin-Ciocalteu (F-C) total phenolics expressed as mg/L gallic acid equivalents.

**Table 3:** Concentrations of wine aroma compounds (expressed as *μ*g/L) related to different yeast strains (BM 4×4, RA17, RC212, WLP77, JH-2, and CR476), Control (Control), and retention index (RI), (*n* = 4).

Number	RI	Compounds	Threshold (*μ*g/L)^A^	Odor descriptor^A^	Odor series^B^	Control	BM 4×4	RA17	RC212	WLP77	JH-2	CR476
1	895	Ethyl acetate	32000.0	Pineapple	1	1971 ± 72^a^	2605 ± 157^bc^	2587 ± 120^bc^	2412 ± 155^b^	2712 ± 142^c^	2307 ± 83^b^	2287 ± 74^b^
2	953	Methyl butyrate	4300.0	Apple, banana, pineapple	1	278 ± 61^a^	262 ± 53^a^	312 ± 49^a^	305 ± 26^a^	254 ± 50^a^	291 ± 38^a^	287 ± 42^a^
3	1018	Isobutyl acetate	365.7	Fruity, grassy, earthy	1, 5, 9	176 ± 29^a^	211 ± 29^ab^	287 ± 41^b^	275 ± 21^b^	189 ± 33^a^	183 ± 17^a^	176 ± 38^a^
4	1037	Ethyl butyrate	20.0	Pineapple, floral	1, 4	136 ± 12^a^	172 ± 23^b^	226 ± 45^c^	238 ± 67^c^	250 ± 35^c^	254 ± 21^c^	282 ± 55^c^
5	1172	Isoamyl acetate	7000.0	Banana	1	1883 ± 96^a^	2060 ± 153^ab^	2127 ± 103^b^	2485 ± 105^c^	2539 ± 86^c^	2787 ± 120^c^	2662 ± 167^c^
6	1225	Ethyl caproate^*∗∗*^	23.0	Fruity, sweet	1, 3	103 ± 34^a^	209 ± 36^b^	313 ± 69^b^	612 ± 134^d^	403 ± 156^cd^	379 ± 73^c^	285 ± 76^b^
7	1248	Hexyl acetate^*∗*^	670.0	Fruity, anise	1, 5	135 ± 21^a^	285 ± 43^c^	137 ± 29^a^	ND	179 ± 35^b^	159 ± 48^ab^	152 ± 23^ab^
8	1413	Ethyl caprylate^*∗*^	580.0	Fruity	1	2155 ± 124^a^	3125 ± 156^c^	2786 ± 187^b^	3312 ± 201^d^	3263 ± 151^cd^	3078 ± 129^c^	2076 ± 110^a^
9	1602	Ethyl decanoate	1122.3	Grape	1	2143 ± 89^a^	2663 ± 104^b^	2200 ± 156^b^	2511 ± 135^b^	2350 ± 97^ab^	2400 ± 113^b^	2139 ± 147^a^
10	1639	Ethyl benzoate^*∗*^	60.0	Fruity	1	86.4 ± 15.0^a^	62.5 ± 12.4^a^	125.1 ± 5.6^bc^	200.8 ± 25.9^c^	37.5 ± 8.2^b^	22.3 ± 6.7^bc^	37.5 ± 10.3^b^
11	1819	Ethyl laurate^*∗∗*^	3500.0	Sweet, fruity	1, 3	ND	265 ± 33^b^	112 ± 22^a^	387 ± 35^c^	350 ± 36^c^	283 ± 43^b^	521 ± 49^d^
12	1974	Ethyl myristate^*∗∗*^	836.2	Iris	4	237 ± 67^a^	165 ± 81^ab^	636 ± 83^c^	162 ± 35^ab^	885 ± 90^d^	389 ± 56^b^	265 ± 73^a^
13	2010	Ethyl cinnamate^*∗∗∗*^	1000.0	Cinnamon, strawberry, honey	1, 3, 6	30.6 ± 6.4^a^	ND	272.3 ± 49.4^d^	88.4 ± 19.7^b^	25.7 ± 11.2^a^	102.0 ± 17.8^c^	114.8 ± 25.4^c^
14	1095	Isobutyl alcohol	7000.0	Ethanol, oil flavor	2, 7	1937 ± 83^a^	2400.3 ± 143.1^b^	2175 ± 98^ab^	2576 ± 85^b^	2461 ± 135^b^	2355 ± 176^b^	2638 ± 71^b^
15	1132	Butyl alcohol^*∗∗*^	500.0	Ethanol, oil flavor	2, 7	18.6 ± 2.1^a^	77.8 ± 23.1^c^	42.5 ± 9.6^b^	150.1 ± 15.4^d^	63.7 ± 12.2^c^	72.3 ± 26.4^c^	37.2 ± 8.3^b^
16	1192	Isopentyl alcohol^*∗*^	300.0	Laurel oil flavor	4	3855 ± 254^a^	6337.6 ± 364.1^c^	5965 ± 469^c^	5965 ± 469^c^	4937 ± 211^b^	5663 ± 369^bc^	6051 ± 298^c^
17	1259	Pentyl alcohol^*∗∗∗*^	6000.0	Fruity, fatty	1, 2	50.3 ± 6.4^a^	24.6 ± 2.4^bc^	126.2 ± 15.7^c^	87.5 ± 9.4^bc^	77.4 ± 12.3^b^	78.1 ± 19.5^b^	136.6 ± 11.0^d^
18	1533	Octyl alcohol^*∗*^	120.0	Fruity	1	4.3 ± 0.8^a^	18.2 ± 9.4^bc^	30.8 ± 10.7^c^	42.6 ± 15.6^d^	16.3 ± 3.2^b^	29.5 ± 9.5^c^	13.3 ± 2.1^b^
19	1900	Benzeneethanol^*∗*^	498.0	Honey, rose	4, 3	121 ± 9^a^	266 ± 35^cd^	451 ± 22^d^	237 ± 20^c^	115 ± 8^a^	257 ± 34^c^	178 ± 15^b^
20	1933	1-Dodecanol^*∗∗*^	2000.0	Floral	4	43.7 ± 10.4^a^	35.3 ± 6.3^a^	27.1 ± 5.2^b^	129.0 ± 11.7^c^	38.6 ± 9.6^a^	39.1 ± 10.0^a^	51.5 ± 13.1^a^
21	2045	Eugenol^*∗*^	30.0	Clove	4	38.9 ± 7.0^a^	25.6 ± 3.6^b^	34.5 ± 4.6^a^	11.7 ± 2.9^c^	27.1 ± 5.3^ab^	19.8 ± 4.2^b^	24.3 ± 5.9^b^
22	1420	Acetic acid^*∗∗*^	327.5	Sour, rancid	2, 8	134.7 ± 19.4^a^	76.6 ± 12.4^b^	88.0 ± 15.3^b^	150.5 ± 22.8^a^	39.1 ± 9.5^c^	30.7 ± 10.1^c^	ND
23	1594	Isobutyric acid^*∗*^	240.0	Sour, rancid	2, 8	89.2 ± 15.7^a^	10.1 ± 3.2^d^	13.4 ± 2.5^d^	57.5 ± 9.4^b^	11.7 ± 2.3^d^	24.2 ± 11.1^c^	39.0 ± 8.6^c^
24	2055	Octanoic acid^*∗*^	2950.0	Unpleasant, cheese	2	1070 ± 122^a^	2510 ± 289^c^	1099 ± 156^a^	1227 ± 189^a^	1335 ± 89^ab^	1573 ± 134^b^	1952 ± 217^bc^
25	2287	Decanoic acid^*∗∗*^	13000.0	Fatty acid	2	830 ± 105^a^	3200 ± 256^d^	1088 ± 133^a^	2289 ± 174^c^	1085 ± 79^a^	1232 ± 71^b^	910 ± 78^a^
26	1376	2-Nonanone	2000.0	Floral	2	19.0 ± 5.6^a^	27.6 ± 4.7^ab^	21.4 ± 5.9^a^	29.1 ± 8.2^ab^	17.6 ± 5.7^a^	32.6 ± 3.5^b^	22.5 ± 9.3^a^

ND: not detected.

Values with different superscript roman letters (a–d) in the same row are significantly different; *∗* represents significance at (*P* < 0.05), *∗∗* at (*P* < 0.01), and *∗∗∗* at (*P* < 0.0001).

^A^Reported odor descriptor and odor threshold come from Gürbüz et al. [34], Lund et al. [36], and Perestrelo et al. [37].

^B^1 = fruity; 2 = fatty; 3 = sweet; 4 = floral; 5 = green; 6 = spicy; 7 = solvent; 8 = sour; 9 = earth.
